# High-Temperature Wear Behaviour of Spark Plasma Sintered AlCoCrFeNiTi_0.5_ High-Entropy Alloy

**DOI:** 10.3390/e21060582

**Published:** 2019-06-12

**Authors:** Martin Löbel, Thomas Lindner, Robert Pippig, Thomas Lampke

**Affiliations:** Materials and Surface Engineering Group, Institute of Materials Science and Engineering, Chemnitz University of Technology, D-09107 Chemnitz, Germany

**Keywords:** high-entropy alloy (HEA), compositionally complex alloy (CCA), spark plasma sintering (SPS), phase composition, microstructure, wear, high temperature

## Abstract

In this study, the wear behaviour of a powder metallurgically produced AlCoCrFeNiTi_0.5_ high-entropy alloy (HEAs) is investigated at elevated temperatures. Spark plasma sintering (SPS) of inert gas atomised feedstock enables the production of dense bulk material. The microstructure evolution and phase formation are analysed. The high cooling rate in the atomisation process results in spherical powder with a microstructure comprising two finely distributed body-centred cubic phases. An additional phase with a complex crystal structure precipitates during SPS processing, while no coarsening of microstructural features occurs. The wear resistance under reciprocating wear conditions increases at elevated temperatures due to the formation of a protective oxide layer under atmospherical conditions. Additionally, the coefficient of friction (COF) slightly decreases with increasing temperature. SPS processing is suitable for the production of HEA bulk material. An increase in the wear resistance at elevated temperature enables high temperature applications of the HEA system AlCoCrFeNiTi_0.5_.

## 1. Introduction

High-entropy alloys (HEAs) present a new approach of alloying concepts. Whereas conventional alloys usually comprise one major alloying element and minor additions of other alloying elements to improve properties, HEAs consist of at least five elements with close to equimolar composition. For several compositions, the desired formation of solely solid solutions with a face-centred cubic (fcc) or a body-centred cubic (bcc) structure could be achieved [1,2,3]. 

Several groups of HEAs have been intensively investigated. The term compositionally complex alloys (CCAs) was introduced for HEAs with a multiphase microstructure [4]. Several investigations prove the formation of primarily cubic phases in the alloy AlCoCrCuFeNi and its derivatives [5,6,7]. Aluminium strongly influences the microstructure, phase formation and resulting properties. Furthermore, aluminium acts as a stabiliser of bcc solid solutions and can suppress the formation of complex phases [8,9,10]. Due to the high atomic radius, alloying with titanium can increase the hardness, the strength and the wear resistance. The highest wear resistance under various conditions, also in comparison with the bearing steel EN 1.3505, could be achieved for the alloy AlCoCrFeNiTi_0.5_ with a medium titanium content. No brittle behaviour under abrasive wear conditions occurred for this alloy, comprising only cubic phases for the as-cast state [11]. Embrittlement was observed for high contents due the formation of complex phases [12,13]. Only few studies have been carried out to examine the wear behaviour at elevated temperature. The sliding wear behaviour in the pin-on-disc test at temperatures of up to 900 °C was investigated [14,15,16]. The reciprocating wear behaviour still needs to be examined. Besides mechanical properties and wear resistance, the magnetic properties of several HEA systems have been investigated revealing interesting properties [17,18].

Primarily, casting has been applied for the production of HEAs. Usually, arc-melting is used, where alloying elements are melted and fully mixed in the liquid state. To achieve chemical homogeneity, the castings are melted several times. However, typical casting defects, e.g., cavities, cracks and elemental segregation, can occur [3,19]. Powder metallurgical processes offer an alternative manufacturing route. Especially, the process of spark plasma sintering (SPS) offers several advantages in comparison to casting processes, but also to other sintering processes. Due to high heating rates and a direct heating of the powder, relatively low sintering temperatures and short process times can be applied. Furthermore, a low porosity and the absence of grain coarsening can be achieved for SPS samples [20,21]. 

The present study focuses on the wear behaviour of the powder metallurgically produced HEA AlCoCrFeNiTi_0.5_. Detailed investigations regarding the microstructure and phase formation of the inert gas atomised feedstock as well as the SPS samples are conducted. The wear behaviour is investigated under reciprocating wear conditions from ambient temperature up to 900 °C. The underlying wear mechanisms are analysed in conjunction with the phase formation.

## 2. Materials and Methods 

Feedstock of the alloy AlCoCrFeNiTi_0.5_ was produced by inert gas atomisation using argon as the process gas. The powder was separated by air classification and a particle size of (−d90 +d10) −45 +20 µm was used. Cross-sections of the powder were prepared by standard metallographic procedures and analysed by scanning electron microscopy (SEM) in a LEO 1455VP (Zeiss, Jena, Germany) with an acceleration voltage of 25 kV and a working distance of 14.5 mm to investigate the morphology and microstructure. A backscattered electron detector (BSE) was used for the visualisation of material contrast. The chemical composition was measured by energy-dispersive X-ray spectroscopy (EDS) with an EDS GENESIS system (EDAX, Mahwah, NJ, USA). Phase analyses were conducted by X-ray diffraction (XRD) with a D8 Discover diffractometer (Bruker AXS, Billerica, MA, USA). For the measurements, Co Kα radiation (U: 40 kV; I: 40 mA), a diffraction angle range (2θ) of 20 to 130°, a 1D Lynxeye XE detector (Bruker AXS, Billerica, MA, USA), point focus and a 1 mm collimator were applied. The assignment of the formed phases was carried out with the powder diffraction file (PDF) database 2014 (International Centre for Diffraction Data). To investigate the particle size distribution, laser diffraction analysis with a Cilas 920 device (Cilas, Orléans, France) was used.

The fabrication of cylindrical samples with a diameter of 40 mm and a height of 6 mm by spark plasma sintering (SPS) was conducted in a SPS KCE FCT-HP D 25-SI (FCT Systeme GmbH, Frankenblick, Germany), equipped with a pyrometer for temperature measurement. Therefore, a graphite die and graphite foil with a thickness of 0.3 mm, placed between powder and punches as a release agent, were used. In order to ensure uniform temperature distribution, the whole graphite tool was thermally insulated. Prior to the sintering process, the recipient was flushed with argon and evacuated (< 1 mbar) twice. After reaching a compaction pressure of 50 MPa, the samples were sintered at a temperature of 1050 °C for 10 min. Subsequently, the samples were cooled passively and unregulated by the water-cooled stamps. This results in a cooling rate of 150 K/min until 300 °C.

For the characterisation of the SPS samples, cross-sections were produced by standard metallographic procedures and analysed by SEM in accordance with powder characterisation. Furthermore, the chemical composition was determined by EDS and phase analyses were conducted by XRD, both in accordance with powder characterisation. The microhardness (Vickers hardness HV0.5) was measured with a Wilson Tukon 1102 device (Buehler, Uzwil, Switzerland). For the calculation of the average value and standard deviation, ten single measurements were considered. The investigation of reciprocating wear behaviour was carried out with an SRV-Tribometer (Optimol Instruments Prüftechnik GmbH, Munich, Germany) under atmospherical conditions. Prior to wear investigations, the temperature was held constant for 5 min. The applied parameters are summarised in Table 1.

For the investigation of the resulting wear depth, a laser scanning microscope (LSM) Keyence VK-X200 (Keyence, Osaka, Japan) was used. Furthermore, the wear tracks were analysed by SEM. To determine the formed oxides within the wear tracks, XRD measurements were conducted after the wear investigations with point focus and a 0.5 mm collimator. The average coefficient of friction (COF) and the standard deviation were determined for all investigations. For the calculation, the test time starting from 60 s until the end of the test was considered.

## 3. Results and Discussion

### 3.1. Feedstock and SPS Sample Characterisation

**Figure 1:**
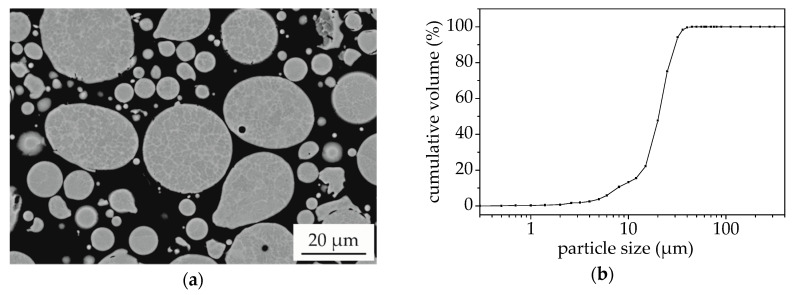
Powder characterisation of AlCoCrFeNiTi_0.5_ feedstock: (**a**) SEM (BSE) cross-section view; (**b**) Cumulative particle size distribution (volume).

The microstructure and morphology of the atomised powder were investigated in cross-sections. The SEM image is shown in Figure 1a. The single particles exhibit a predominantly spherical shape. A dendritic structure with distinct BSE contrast can be observed, indicating a deviating chemical composition. Due to the high cooling rates during the atomisation process and hence fast solidification, a microstructure with a finer distribution of the phases occurs in comparison to arc-melted samples of the same alloy [11].

**Figure 2:**
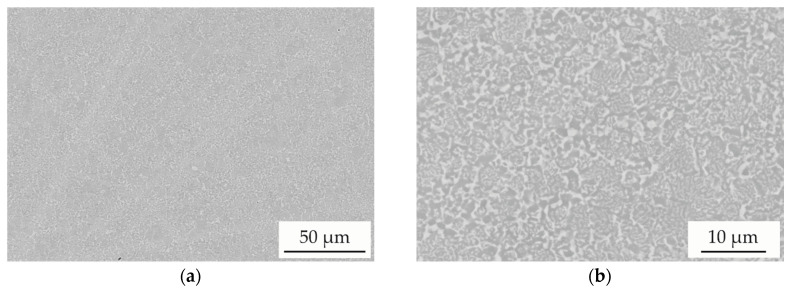
SEM images (BSE) of AlCoCrFeNiTi_0.5_ samples produced by spark plasma sintering (SPS): (**a**) Overview; (**b**) In detail.

The particle size distribution is shown in Figure 1b. A mean particle size (d50) of 20 µm and a particle size range (−d90 +d10) of −30 +8 µm was determined, showing that despite air classification a high content of very fine particles with a diameter below 20 µm is present in the feedstock. The atomised powder was applied for the production of SPS samples with the mentioned parameters. SEM images of the cross-sections with different magnification are shown in Figure 2.

The overview image shows that a dense structure without porosity is produced. In comparison to single powder particles, the SPS samples exhibit a similar microstructure with a distinct deviation of chemical composition. No significant coarsening of microstructural features occurs during SPS processing. A good correlation between the average chemical composition of the powder and SPS samples was found. The results of the EDS measurements in comparison to the nominal values are shown in Table 2.

**Figure 3:**
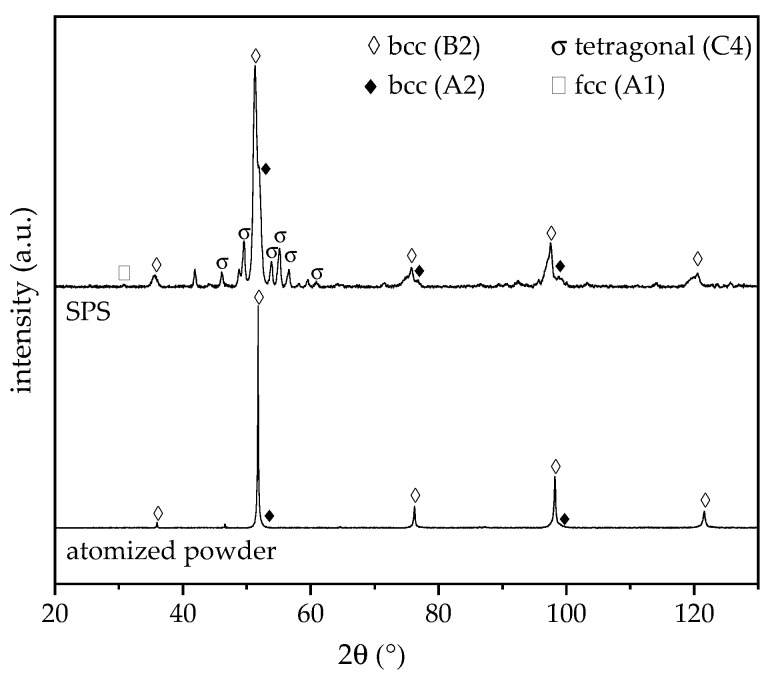
XRD diffractograms of AlCoCrFeNiTi_0.5_ atomised powder and resulting SPS samples.

For the atomised powder, only the aluminium content shows a deviation exceeding 1 at.%. Processing of the powder by SPS does not cause a significant change in the chemical composition. The resulting diffractograms of the XRD phase analyses for the feedstock and SPS samples are displayed in Figure 3. The diffractograms were scaled to uniform intensity of the maxima.

In accordance with the feedstock, the SPS samples exhibit a multiphase microstructure. However, additional phases appear after SPS processing. This is in accordance to the results of previous investigations on arc-melted samples [11].

The diffractogram of the atomised powder exhibits major diffraction peaks of a chemically ordered bcc phase with B2 structure. A shoulder towards higher diffraction angles can be observed for the intensity maxima at 51.8° and 98.2°, indicating the presence of a second phase. Microstructural investigations also suggest a multiphase structure due to a strong material contrast between different areas. An overlapping with a chemically disordered bcc phase with A2 structure occurs. Previous investigations on arc-melted samples revealed the formation of the bcc phase with B2 structure as the primary phase, whereas the bcc phase with A2 structure precipitates subsequently as the interdendritic phase [11]. For the SPS samples, the diffraction peaks of the two bcc phases with A2 or B2 structures can be observed. The intensity maxima of the chemically ordered bcc phase with B2 structure are slightly shifted to lower diffraction angles in comparison to the feedstock powder. This behaviour indicates an increase in the lattice parameter due to SPS processing. Furthermore, a minor diffraction peak occurs at 30.8°, which is characteristic for an fcc phase with A1 structure. All other intensity maxima of this phase overlap with the bcc B2 phase. Due to the fast cooling in the atomisation process, decomposition is suppressed. Additional diffraction peaks can be assigned to a tetragonal σ-phase. This phase was also detected by Moravcik et al. for the alloy AlCoCrFeNiTi_0.5_ produced by SPS. However, a subsequent heat treatment and relatively fast air cooling resulted in the dissolution of the σ-phase [22]. For the SPS sample, an overall hardness of 750 ± 17 HV0.5 was measured. In comparison to the same alloy produced by arc-melting, a distinct increase in the hardness occurs due to the formation of the additional σ-phase and a microstructure with a finer phase distribution [11].

### 3.2. Wear Investigations

**Figure 4:**
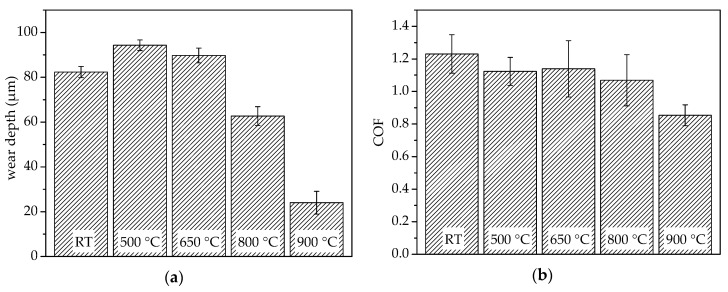
Results of the reciprocating wear investigations for AlCoCrFeNiTi_0.5_ produced by SPS in a temperature range from room temperature (RT) to 900 °C: (**a**) Wear depth; (**b**) Average coeffecient of friction (COF).

An influence of the test temperature on the wear behaviour under reciprocating wear conditions was observed. After completing the tests, the wear depth and average COF were determined. The results from ambient temperature up to 900 °C under atmospherical conditions are summarised in Figure 4. With an increase in temperature from room temperature to 500 °C, a slight increase in the wear depth occurs, indicating a reduced wear resistance. A further increase in test temperature results in a slight decrease in the wear depth. For the wear investigations at 800 °C, a distinct reduction in wear depth occurs. The highest wear resistance was proven for the test conducted at the highest temperature of 900 °C.

Furthermore, the influence of the temperature on the average COF was investigated. With an increase in temperature up to 800 °C, the COF only slightly decreases. A high standard deviation occurs for all of these measurements. The lowest COF was measured for the highest test temperature of 900 °C, with an average value of 0.85.

**Figure 5:**
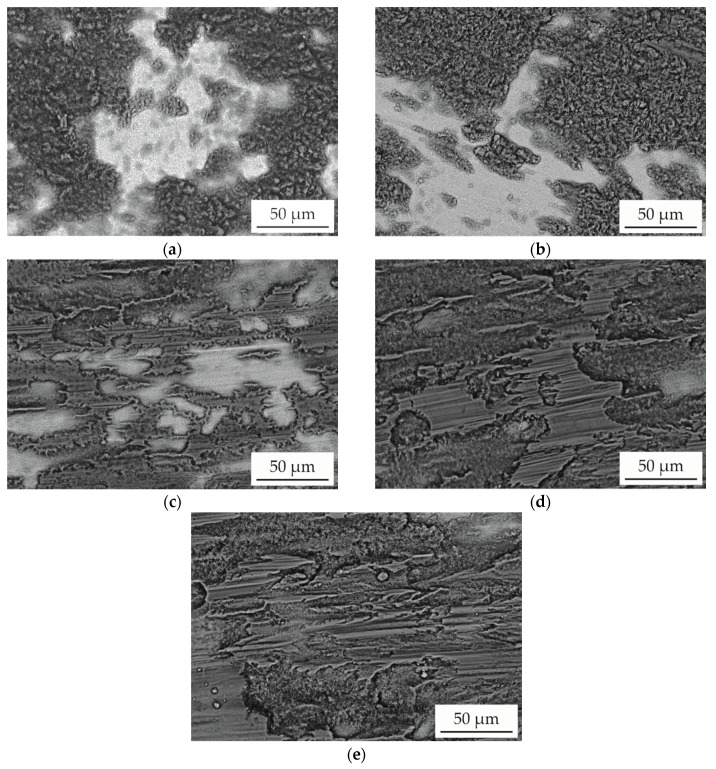
SEM images (BSE) of the AlCoCrFeNiTi_0.5_ SPS sample surface after reciprocating wear test at a temperature of: (**a**) room temperature; (**b**) 500 °C; (**c**) 650 °C; (**d**) 800 °C; (**e**) 900 °C.

To investigate the underlying wear mechanisms, detailed investigations of the wear tracks were conducted. SEM images (BSE detector) of the worn area are shown in Figure 5. A strong material contrast can be observed. Dark-appearing areas indicate a high concentration of elements with a low atomic number and hence oxides. The bright-appearing areas can be assigned to the metallic substrate. The surfaces of the samples tested at room temperature and 500 °C are partially covered with oxides. For both test conditions, rough and inhomogeneous oxides are formed, showing that a continuous formation and removal of oxides occur. The surface of the sample tested at a temperature of 650 °C is also incompletely covered with oxides. However, the oxides are less rough and exhibit grooves in wear direction. This behaviour indicates an increasing stability caused by a rise in thickness and hence a protection of the underlying metallic material. Due to the formation of an interrupted oxide layer, a high standard deviation of the COF occurs. With a further increase in the test temperature, the content of the surface covered with oxides gradually increases. For the samples tested at 800 °C and 900 °C, an almost fully oxide-covered surface can be observed, which is relatively smooth and exhibits some grooves. The decrease in wear depth proves the protection of the underlying material by tribo-oxidation and the formaton of a stable oxide film. Furthermore, the appearance indicates abrasive wear of the oxides.

**Figure 6:**
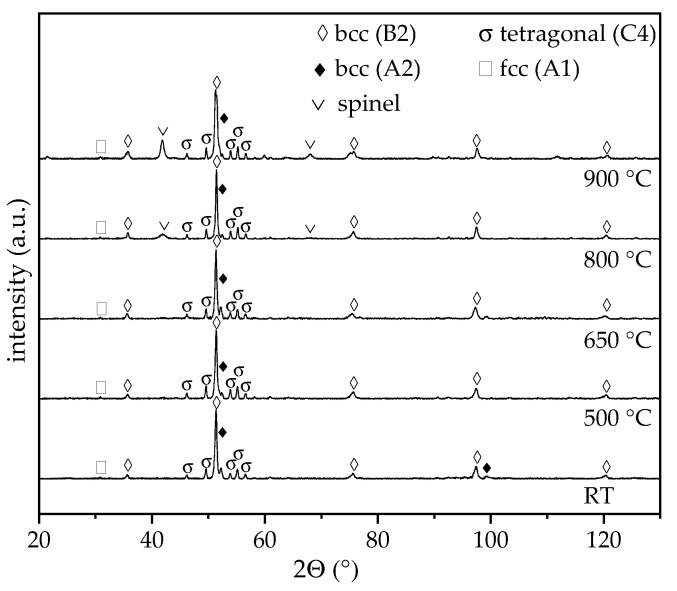
XRD diffractograms of AlCoCrFeNiTi_0.5_ SPS samples after reciprocating wear tests at various temperatures.

For the investigation of the phase constitution within the wear tracks, additional XRD measurements were conducted. The resulting diffractograms are shown in Figure 6. In comparison to the unaffected state (Figure 3), additional diffraction peaks can only be observed for the two highest test temperatures. This behaviour confirms an increased oxide-layer thickness, which is in accordance with the results of the wear tests. The additional maxima can be assigned to a spinel (structure type MgAl_2_O_4_). The intensity of the diffraction peaks increases for the sample tested at a maximum temperature of 900 °C. Furthermore, an additional intensity maximum occurs at 59.9°, which could not be assigned. No diffraction peaks of other oxides were detected.

The results of the phase analyses differ in comparison to previous investigations on the oxidation behaviour of the alloy system AlCoCrFeNiTi. Wang et al. revealed the formation of mainly TiO_2_ and (Al_0.9_Cr_0.1_)_2_O_3_ at an oxidation temperature of 900 °C. An increase in temperature to 1100 °C caused the formation of spinel, TiO_2_, Cr_2_O_3_ and (Al_0.9_Cr_0.1_)_2_O_3_ [23]. The significantly longer duration time of 100 h at higher temperatures increases the oxide-layer thickness and enhances the traceability. Furthermore, differences can be explained by time dependency of phase transformations.

## 4. Summary

Spark plasma sintering (SPS) of AlCoCrFeNiTi_0.5_ was conducted as an alternative powder metallurgical production technique for high-entropy alloys (HEA). A dense microstructure of the sintered parts can be achieved. In comparison to the casting route, the fast cooling rate during atomisation suppresses the segregation of the melt and hence the precipitation of an fcc phase. The samples produced by SPS exhibit a multiphase microstructure comprising two major bcc phases. An ordered bcc phase with B2 structure and a disordered bcc phase with A2 structure are formed. In accordance with samples produced by arc-melting, a minor fcc phase is detected. Furthermore, an additional tetragonal σ-phase was detected after processing by SPS. Due to the formation of the additional phase and a microstructure with a fine phase distribution, a high microhardness of 750 ± 17 HV0.5 was determined. The wear behaviour under reciprocating wear conditions was investigated from room temperature to 900 °C. With increasing temperature, initially a slight decrease in the wear resistance occurred up to a temperature of 650 °C. A further increase in test temperature resulted in a distinct increase in the wear resistance. Detailed investigations of the wear tracks revealed the formation of a protective oxide film and abrasive wear behaviour. Phase analyses proved the formation of a spinel phase. Furthermore, the COF decreases for the measurements at elevated temperatures.

The investigations prove the suitability of AlCoCrFeNiTi_0.5_ for high temperature applications. Due to the formation of protective oxides, an improvement of wear behaviour occurs. Further investigations have to be conducted to determine the wear behaviour under different wear conditions. The influence of the additional σ-phase has to be investigated in subsequent studies.

## Figures and Tables

**Table 1:** Wear test parameters.

Reciprocating Wear Test	
Force	26 N
Frequency	40 Hz
Time	900 s
Amplitude	0.5 mm
Counter Body	Al_2_O_3_
Diameter	10 mm
Temperature	22 °C; 500 °C; 650 °C; 800 °C; 900°C

**Table 2:** Nominal values and measured chemical composition of the atomised powder and SPS sample in at.%.

Sample	Nominal	Atomised Powder	SPS
Al	18.2	20.1	21.0
Co	18.2	17.5	17.3
Cr	18.2	18.0	17.7
Fe	18.2	17.9	17.5
Ni	18.2	17.5	17.5
Ti	9.1	9.0	8.9
